# Novel prognostic gene signature for pancreatic ductal adenocarcinoma based on hypoxia

**DOI:** 10.1186/s12957-023-03142-2

**Published:** 2023-08-22

**Authors:** Min Ren, Liaoliao Feng, Rongrong Zong, Huiru Sun

**Affiliations:** College of Life Science, Yan’an University, Yan’an, 716000 China

**Keywords:** Gene signature, Prognosis, Hypoxia, Pancreatic ductal adenocarcinoma (PDAC), ARID5A

## Abstract

**Background:**

Currently, there is lack of marker to accurately assess the prognosis of patients diagnosed with pancreatic ductal adenocarcinoma (PDAC). This study aims to establish a hypoxia-related risk scoring model that can effectively predict the prognosis and chemotherapy outcomes of PDAC patients.

**Methods:**

Using unsupervised consensus clustering algorithms, we comprehensively analyzed The Cancer Genome Atlas (TCGA) data to identify two distinct hypoxia clusters and used the weighted gene co-expression network analysis (WGCNA) to examine gene sets significantly associated with these hypoxia clusters. Then univariate Cox regression, the least absolute shrinkage and selection operator (LASSO) Cox regression and multivariate Cox regression were used to construct a signature and its efficacy was evaluated using the International Cancer Genome Consortium (ICGC) PDAC cohort. Further, the correlation between the risk scores obtained from the signature and carious clinical, pathological, immunophenotype, and immunoinfiltration factors as well as the differences in immunotherapy potential and response to common chemotherapy drugs between high-risk and low-risk groups were evaluated.

**Results:**

From a total of 8 significantly related modules and 4423 genes, 5 hypoxia-related signature genes were identified to construct a risk model. Further analysis revealed that the overall survival rate (OS) of patients in the low-risk group was significantly higher than the high-risk group. Univariate and multivariate Cox regression analysis showed that the risk scoring signature was an independent factor for prognosis prediction. Analysis of immunocyte infiltration and immunophenotype showed that the immune score and the anticancer immune response in the high-risk were significantly lower than that in the low-risk group.

**Conclusion:**

The constructed hypoxia-associated prognostic signature demonstrated could be used as a potential risk classifier for PDAC.

**Supplementary Information:**

The online version contains supplementary material available at 10.1186/s12957-023-03142-2.

## Introduction

Pancreatic ductal adenocarcinoma (PDAC) is a highly lethal malignancy affecting the devastating digestive tract, characterized by high mortality [1]. Due to the absence of early symptoms and reliable early detection methods, most PDAC cases are diagnosed at an advanced stage, resulting in a very poor 5-year overall survival rate (< 10%) [2, 3]. Despite advancements in therapies such as chemoradiotherapy and immunotherapy, there has been limited improvement in the survival rates of PDAC patients [4, 5]. Currently, surgical excision remains the only radical treatment option, with only 20% percent of patients eligible for curative surgery [6].

An early diagnosis of cancer and early intervention for “high-risk” patients are critical to improving the clinical outcomes of cancer patients. Currently, predictive models based on cumulative clinical and pathological parameters, such as the American Joint Committee on Cancer (AJCC) grading system, which is based on the tumor-node-metastasis (TNM) classification, are commonly used to assess the prognosis of cancer patients [7]. However, this system remains unsuitable for the individualized treatment of PDAC patients because it is difficult to reflect their molecular profile and lack the ability to adjust the staging as the disease progresses [8]. Furthermore, this classification system may result potential undertreatment or overtreatment.

Surgical resection combined with chemoradiotherapy remains the most widely utilized treatment option for PDAC, despite only providing modest improvements in survival rates [9–11]. In recently years, immunotherapy targeting immune checkpoint inhibitors (ICIs) has emerged as a promising treatment option for various cancers [12]. Biomarkers such as programmed death-ligand 1 (PD-L1) expression, tumor mutation burden (TMB), neoantigen load (NAL), and microsatellite instability-high (MSI-H) have been identified as potential biomarkers for the effectiveness of ICIs [13–15]. While immunotherapy alone has shown limited efficacy in pancreatic cancer, the combination of ICIs with chemoradiotherapy has demonstrated promising results [16]. Consequently, the identification of related reliable prognostic and therapeutic biomarkers is crucial for precise and individualized precise treatments for PDAC patients.

Hypoxia is a major feature of PDAC [17]. Although tumor cells can deplete oxygen levels in the microenvironment through rapid cell proliferation and altered metabolism, they tend to exhibit a remarkable tolerance to hypoxia, contributing to tumor progression and treatment resistance [18–20]. PDAC is typically characterized by a median oxygen level of less than 0.7%, along with the activation of angiogenesis and glycolysis pathways within the hypoxic microenvironment [21, 22]. Numerous studies have indicated that tumor hypoxia is associated with anti-apoptosis, tumor recurrence, migration, immunosuppression, immune evasion, and resistance to chemoradiotherapy [19, 22–25], further confirming that hypoxic status is significantly correlated with the prognosis of PDAC.

**Fig. 1:**
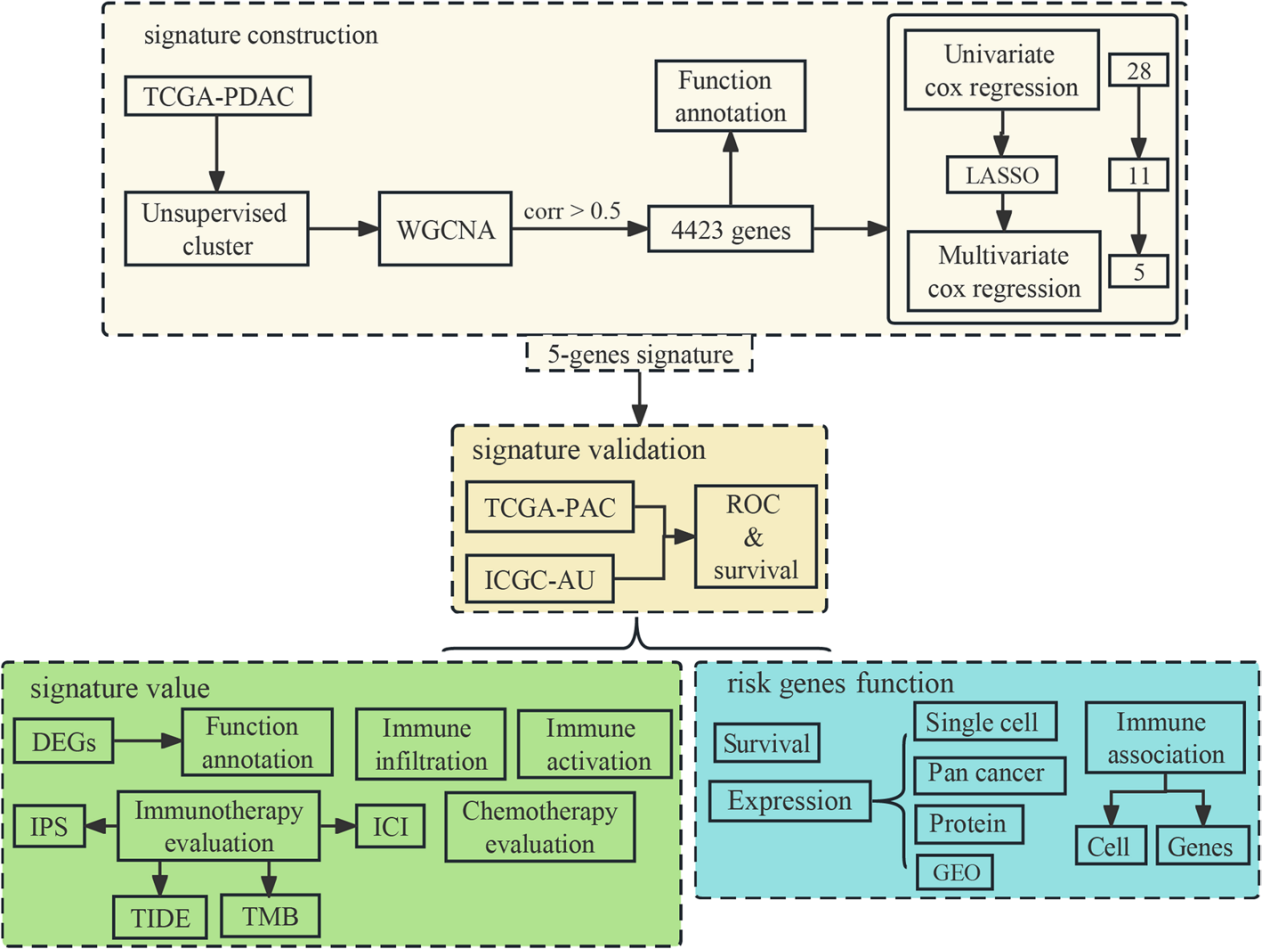
Flowchart for the development and validation of a PDAC prognostic signature based on hypoxia

In this study, we utilized the TCGA and ICGC databases to develop and validate a prognostic signature for PDAC based on hypoxia-related gene sets and further assessed the predictive ability of the model for both immunotherapy and chemotherapy (Fig. 1). The results showed that our developed signature may serve as a valuable supplement to the existing clinical staging system, enabling early detection and intervention for individuals at high-risk patients and improving their’ prognosis.

## Materials and methods

### Data collection and preparation

Transcription expression profiles of PDAC patients in TCGA and ICGC databases were obtained from the XENA platform (https://xenabrowser.net/datapages, 2022) [26]. The expression values were quantified using the fragments per kilobase of exon per million reads mapped (FPKM) method and then transformed using log_2_(X + 1). Non tumor samples were excluded from the analysis. Hypoxia hallmark genes (*n* = 200) were obtained from the Molecular Signatures Database (MsigDB, https://www.gsea-msigdb.org/gsea/msigdb/). Somatic mutation data for PDAC patients from the TCGA dataset were acquired from the Genomic Data Commons (GDC, https://portal.gdc.cancer.gov/, 2022). The clinical information for the TCGA dataset is provided in Table 1. All analyses were performed using the R language (version 4.2.2).

**Table 1:** Clinical information of included PDAC patients in TCGA

Characteristics	Level	Samples (percentage)
Total		141
Status (%)	Alive	60 (42.6)
	Dead	81 (57.4)
Age (%)	Old	70 (49.6)
	Young	71 (50.4)
Gender (%)	Female	67 (47.5)
	Male	74 (52.5)
Tumor_stage (%)	I	13 (9.2)
	II	121 (85.8)
	III/IV	6 (4.3)
	NA	1 (0.7)
N.stage (%)	N0	37 (26.2)
	N1	103 (73.0)
	NA	1 (0.7)
T.stage (%)	T1/T2	21 (14.9)
	T3/T4	119 (84.4)
	NA	1 (0.7)
M.stage (%)	M0	65 (46.1)
	M1	3 (2.1)
	MX	73 (51.8)
Tumor site (%)	Head	107 (75.9)
	Other	34 (24.1)
Malignancy (%)	Malignancy	9 (6.4)
	Not	132 (93.6)
Grade (%)	G1/G2	100 (70.9)
	G3/G4	40 (28.4)
	NA	1 (0.7)

### Unsupervised clustering of hypoxia genes

For the clustering analysis, we employed the R package ConsensusClusterPlus (version 1.60.0) to cluster the expression profiles of PDAC patients in TCGA based on the hypoxia-related gene set obtained from MSigDB. The clustering process involved performing 1000 iteration, each utilizing 80% of the data. The optimal number of clusters was determined by evaluating the Consensus–Matrix plot and the relative change in the area under the cumulative distribution function (CDF) curves. Ultimately, two clusters were selected to represent different hypoxia statues. To assess the impact of these clusters on patients’ survival, Kaplan–Meier (K-M) plots were generated using the survival package (version 3.5–5). K-M plots were generated to compare their overall survival (OS) of patients in cluster_1 and cluster_2. Additionally, the R package progeny (version 1.18.0) was used to calculate the activation scores of 14 typical pathways in the TCGA-PDAC cohort [27], and the Wilcoxon signed-rank test was employed to assess differences in pathway activation between cluster_1 and cluster_2 groups.

### Weighted gene co-expression network analysis for hypoxia clustering

The R package WGCNA (version 1.72.1) was used to investigate co-expression networks and identify gene sets associated with hypoxia status in the TCGA-PDAC cohort [28]. First, we calculated the optimal soft threshold β that met the criteria for a scale-free network, employed a one-step method to build the co-expression network, and set the minimum number of genes within each module to 40 to ensure robust modules. Additionally, module fusion was performed, setting the height of dendrogram cutting for module merging at 0.1. Module eigengenes (MEs) were generated by summarizing the first principal component of each module’s gene expression. Person’s correlation coefficient between MEs and the hypoxia status clusters were evaluated. Modules with correlation coefficients greater than 0.5 were selected for further analysis. The genes within these modules were considered candidate genes for further analysis. Lastly, Kyoto Encyclopedia of Genes and Genomes (KEGG) and Gene Ontology (GO) functional enrichment analyses were performed on the candidate genes using the R package clusterProfiler (version 3.16.1) [29].

### Identification of prognostic genes and construction risk signature

The TCGA-PDAC cohort was selected as the training set for constructing the hypoxia-related risk model, while the ICGC cohort served as the validation set, and jointed Cox regression analysis was performed to construct the prognostic model. First, univariate Cox regression was performed to identify genes significantly associated with OS from the candidate gene set using R package Survival. Then, the least absolute shrinkage and selection operator (LASSO) penalized Cox regression model was applied to analyze the genes with *p* < 0.001 to reduce the risk of overfitting using the glmnet package (version 4.1–4) [30, 31]. Lastly, multivariate Cox regression and stepwise method were used to identify independent genes for building the prognostic model and their regression coefficients using the Survival package. The risk score of the patients was calculated according to the normalized expression of each gene and its coefficient and calculated using the following formula:

$$\text{risk score }(\mathrm{patient})=\sum\limits_{k=1}^{n} \left(\mathrm{coef}\times \mathrm{exp}\right)$$

where *n* represents all cases, exp indicates the expression level for each risk gene, and the coef indicates their regression coefficients. Then, the patients were stratified into a high- or low-risk groups using the median risk score.

### Evaluation and verification of the prognostic signature

The difference in OS between the two risk groups was estimated using K-M curves and assessed using the log-rank test by Survival. Receiver operating characteristic curves (ROC) based on the patients’ risk scores were plotted suing the package timeROC (version 0.4) and ggplot2 (version 3.3.6). The concordance index (C index) was calculated to evaluate the prediction accuracy of the prognostic signature by R package survcomp (version 1.46.0) [32]. Univariate and multivariate cox regression analyses were performed on the TCGA-PDAC cohort to verify the clinical predictive independence of the risk score model.

### Differentially expression analysis between risk groups

The R package progeny was used to calculate the activation of 14 typical pathways in the TCGA-PDAC cohort [27]. Wilcoxon signed-rank test was used to detect differences in activation score between the high- and low-risk groups. To explore the differentially expressed genes (DEGs) between the high- and low-risk groups in the TCGA-PDAC cohort, the R package DEseq2 (version 1.36.0) was employed. DEGs were identified based on criteria as |Log Fold change (FC)|> 1 and *p*-value < 0.01 [33]. Pathway analysis was performed using Metascape platform (http://metascape.org/gp/index.html#/main/step1) [34]. The mutation landscape of patients in the TCGA-PDAC cohort was analyzed using the R package maftools (version 2.12.0) [35].

### Estimation of immune cell infiltration fractions

To investigate the differences in the immune microenvironment composition between the high- and low-risk groups in the TCGA-PADC cohort, various methods were employed. Tumor purity and infiltrating immune/stromal cell scores were predicted using the package estimate (version 1.0.13). Single-sample gene set enrichment analysis (ssGSEA) was performed to quantitatively measure immune cell infiltration using the expression profiling matrix from the R package GSVA (version 1.36.3) [36, 37]. As a supplement, all patients’ immune cells proportion based on TIMER, CIBERSORT, XCELL, QUANTISEQ, MCPcounter, EPIC, and CIBERSORT-ABS were downloaded from the TIMER2.0 dataset (http://timer.comp-genomics.org/) [38–44]. Wilcoxon signed-rank test was used to detect differences in immune cell composition between the high- and low-risk groups. Spearman correlation was then used to calculate the correlation between the risk values and the immune cells that were significantly different after the Wilcoxon test.

The activation of the cancer immune cycle, which reflects the antitumor immune response and influences the fate of tumor cells, was obtained for the TCGA-PDAC cohort from Tracking Tumor Immunophenotype database (TIP, http://biocc.hrbmu.edu.cn/TIP/index.jsp). The Wilcoxon signed-rank test was employed to identify pathways that displayed differential activation between the high- and low-risk groups [45].

### Immunotherapy and chemotherapy response predictions

Multiple perspectives were used to assess immunotherapy efficacy in the different risk groups from the TCGA-PADC cohort. The immunophenotype (IPS) of all patients, obtained from the Cancer Immunome Atlas database (TCIA, https://tcia.at/home), was calculated by analyzing the expression genes associated with immunogenicity [46]. The TIDE (http://tide.dfci.harvard.edu/) online algorithm was used for immune checkpoint blockade therapy response predictions [47]. Tumor mutation burden (TMB) and microsatellite instability (MSI) have shown superior ability in predicting immunotherapy response in various cancers [48–50]. TMB of TCGA-PDAC patients was calculated using the package maftools [35]. The neoantigens were downloaded from TCIA [46]. We downloaded the MSI prediction results of TCGA-PDAC patients based on the mantis method [51]. We also used the R package PreMSIm (version 1.0) to predict the MSI for TCGA-PDAC patients [52]. Furthermore, we calculated Spearman correlations between the expression levels of 20 common immune checkpoint genes and risk scores. Additionally, the differences in expression levels of these between the high- and low-risk groups were analyzed using the Wilcoxon signed-rank test.

The half-maximal inhibitory concentration (IC50) values of common PDAC chemotherapy drugs (paclitaxel, erlotinib, gemcitabine, sorafenib, cisplatin, 5-fluorouracil) in each TCGA-PDAC sample were estimated based on the transcription data by R package pRRophetic (version 0.5) and Genomics of Drug Sensitivity in Cancer (GDSC) database (https://www.cancerrxgene.org) [53, 54].

### Functional analysis of risk genes

We used the Wilcoxon signed-rank test to analyze the difference in the expression of risk genes in the high- and low-risk groups. The ssGSEA method was used to estimate the contents of 28 typical immune cells in the TCGA-PADC cohort, and the Spearman correlation coefficients among risk genes were calculated. The expression of risk genes among different cell types was examined using The Human Protein Atlas (HPA, https://www.proteinatlas.org/) and The Tumor Single-Cell Hub (TISCH, http://tisch.comp-genomics.org/home/., the PAAD_GSE154778 dataset was selected) [55, 56]. The risk genes protein distribution information of cancerous and normal pancreatic tissues (Genotype-Tissue Expression database) were obtained from the HPA. Pan-cancer expression analysis of risk genes was performed via the Gene Expression Profiling Interactive Analysis (GEPIA2, http://gepia2.cancer-pku.cn/#index, choice match with TCGA normal and GTEx data) [57]. Pan-cancer survival analysis results of risk genes were obtained from HPA. We used the Gene Expression database of Normal and Tumor tissues (GENT2, http://gent2.appex.kr/gent2/) and the differential expression analysis in Tumor, Normal, and Metastatic tissues (TNMplot, https://tnmplot.com/analysis/) to detect the expression difference of risk genes in multiple pancreatic cancer microarray dataset between tumor and normal samples [58, 59]. In addition, the package LinkET (version 0.0.7.2) was used to detect the association between risk genes and immune activation/suppressor genes.

## Results

### Identification of genes associated with hypoxia in PDAC

**Fig. 2:**
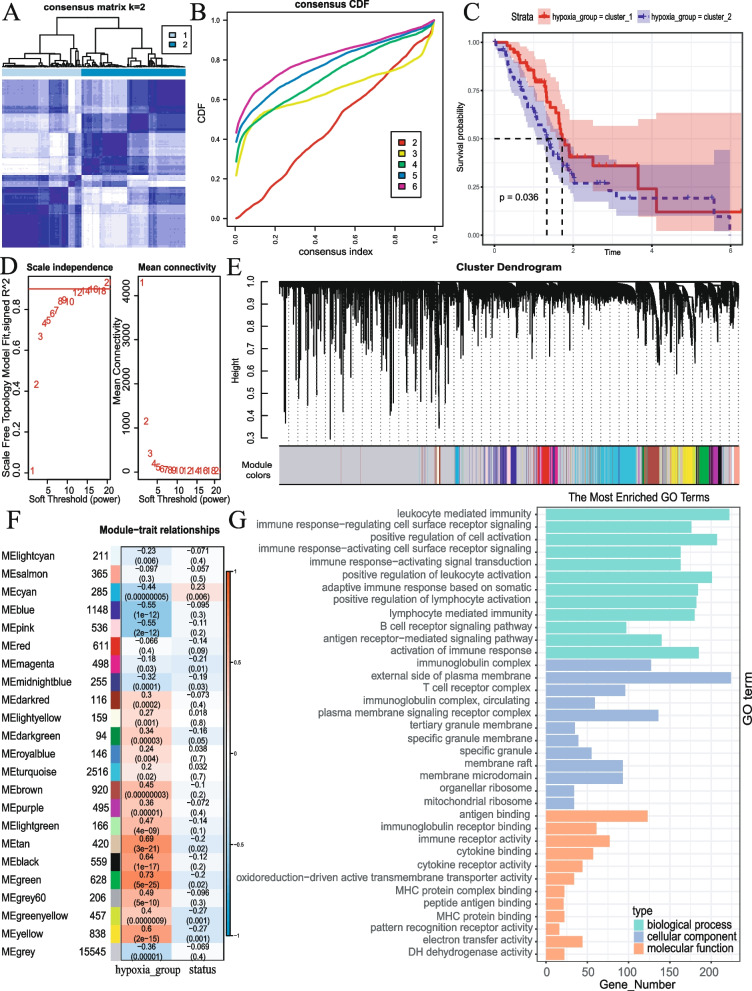
Identification of genes associated with hypoxia in PDAC based on WGCNA. **A, B** The Consensus–Matrix plot and CDF curves were used to confirm the optimal cluster number. **C** Kaplan–Meier curve of OS of PDAC patients in cluster_1 and cluster_2 groups. **D** The optimal evaluating results about soft threshold β. **E** Clustering dendrograms showing genes with similar expression patterns were clustered into co-expression modules. **F** Module–trait relationships revealing the correlations between each gene module eigengene and hypoxia cluster and survival status. **G** Results of GO pathway analysis of candidate gene set

After excluding samples with normal tissues and lacking survival information, 141 and 92 cases were retrieved from the TCGA-PDAC and ICGC-PDAC database. Using the expression matrix comprised of hypoxia-related genes, we performed unsupervised cluster analysis on the PDAC samples. The CDF curves revealed that the optimal number of clusters was achieved when *k* = 2 (Fig. 2A, B). The K-M curve demonstrated a significant difference in OS between the two clusters (Fig. 2C). Moreover, the analysis of pathway activation indicated a significant disparity in the activation degree of the hypoxia pathway between the two clusters, suggesting a significant correlation between our clustering and hypoxia (Fig. S1).

During the WGCNA procedure, the optimal soft threshold β was set to 12 (Fig. 2D) to provide an appropriate suitable power value for the construction of the coexpression network (Fig. 2E); 4423 genes (8 modules) that exhibited significantly association with the hypoxia cluster (Fig. 2F, Fig. S2). GO and KEGG pathway enrichment analysis showed that these genes were mainly related to immune response (Fig. 2G, Fig. S3).

### Construction of prognostic signature related to hypoxia

**Fig. 3:**
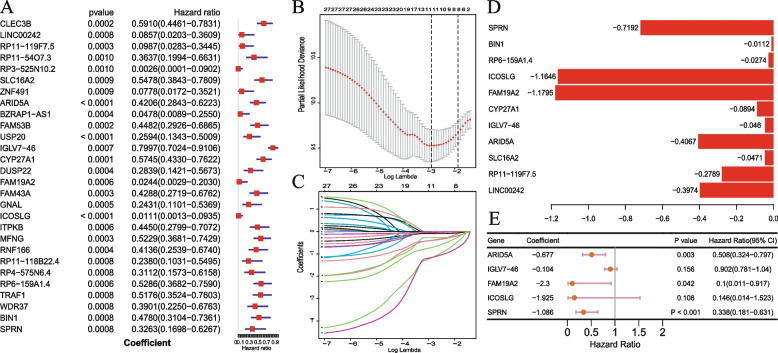
Construction of prognostic signature related to hypoxia. **A** Forest plot of hazard ratios of 28 prognostic genes correlated with OS. **B** Tuning parameter selection in the LASSO model. **C** LASSO coefficient profiles of the 11 genes. **D** 11 genes coefficient after LASSO analysis. **E** Final 5 genes forest plot after multivariate Cox regression

Based on the expression profiles of the 4423 hypoxia-related genes, 28 prognostic genes were identified by univariate Cox analysis (Fig. 3A) and following LASSO Cox analysis, 11 genes were identified after 1000 iterations (Fig. 3B, C, D). Finally, multivariate Cox regression analysis was used to obtain a 5-gene hypoxia-related prognostic signature (Fig. 3E), and the risk score was estimated as follows:

$$\mathrm{Risk}\;\mathrm{score}=\left(-\:0.677\;\ast\;\mathrm{expr}\;\mathrm{of}\;\mathrm{ARID}5\mathrm A\right)+\left(-\:0.104\;\ast\;\mathrm{expr}\;\mathrm{of}\;\mathrm{IGLV}7-46\right)+\left(-\:2.3\;\ast\;\mathrm{expr}\;\mathrm{of}\;\mathrm{FAM}19A2\right)+\left(-\:1.925\;\ast\;\mathrm{expr}\;\mathrm{of}\;\mathrm{ICOSLG}\right)+\left(-\:1.086\;\ast\;\mathrm{expr}\;\mathrm{of}\;\mathrm{SPRN}\right).$$

### Validation of prognostic signature related to hypoxia

**Fig. 4:**
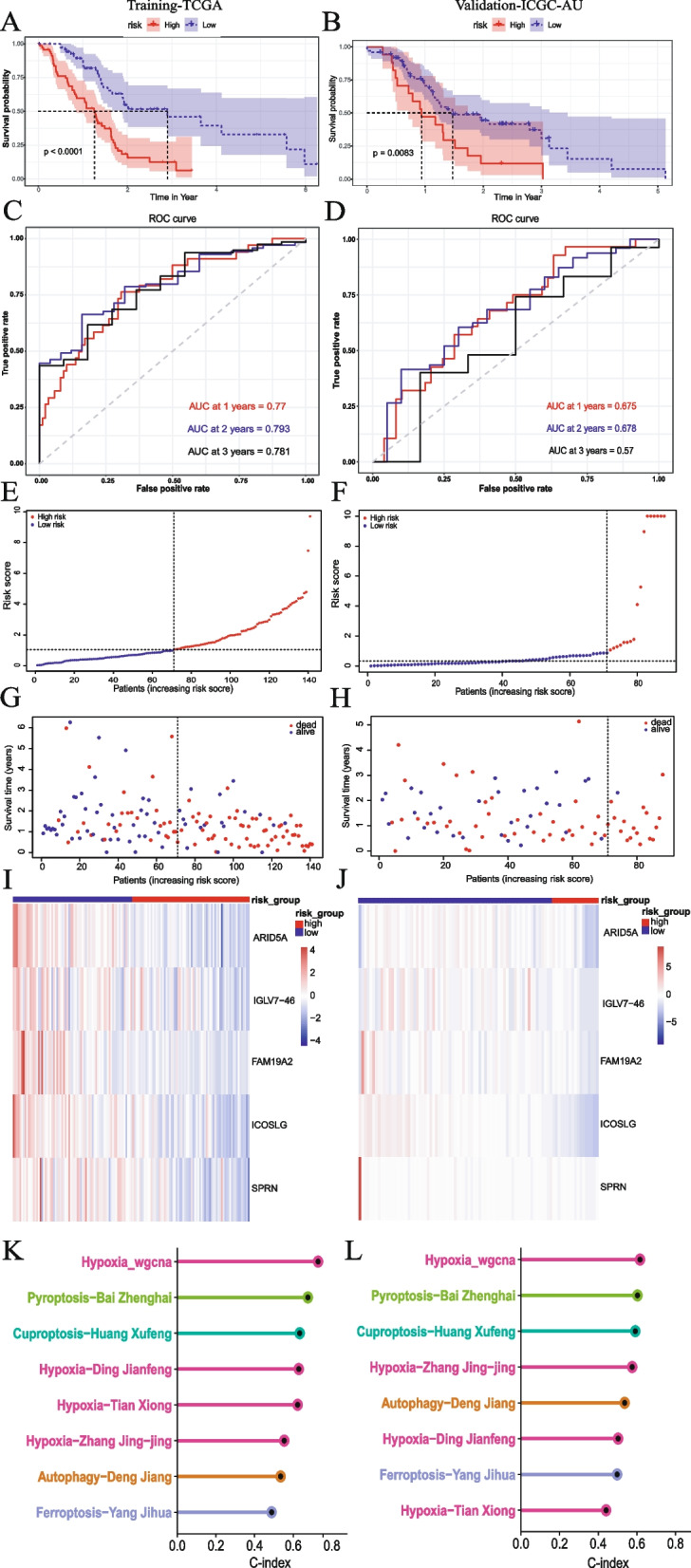
Five genes hypoxia status-related signature evaluation and validation. **A, B** Kaplan–Meier curve of OS of PDAC patients in high- and low-risk groups. **C, D** The 1-, 2-, and 3-year AUC in ROC analysis. **E–H** Risk curve for the investigated patient. **I, J** Heatmap for the expression levels of 5 risk genes. **K, L** The C-index of our signature (Hypoxia_wgcna), 3 published hypoxia-related prognostic signatures, and 4 published cell death signatures

After dividing all patients into high- and low-risk groups based on the median of risk scores, the K-M survival curve demonstrated that the OS in the high-risk group was significantly lower than in the low-risk group in both the training set (*P* < 0.001, Fig. 4A) and validation set (*P* = 0.0083, Fig. 4B). Moreover, a higher frequency of death events was observed in the high-risk group compared to the low-risk group (Fig. 4G, H). The ROC curve analysis in training and validation sets indicated the promising prognostic value of the signature for predicting PDAC OS (1, 2, 3 years AUC > 0.75 in training sets, Fig. 4C. 1, 2 years AUC > 0.65 in validation sets, Fig. 4D). Additionally, as the risk score increased, we observed that the patients’ prognosis worsened in both sets (Fig. 4E, G for training sets, Fig. 4F, H for validation sets). Further, the heatmap showed that the expression of risk genes was higher in the low-risk group (Fig. 4I for training sets, Fig. 4J for validation sets).

To evaluate the prediction accuracy of the prognosis signature, we calculated the C-index for all published hypoxia-related prognostic signatures in PDAC, whereby a C-index greater than 0.7 is considered indicative of an accurate model. Additionally, we compared the predictive performance of our signature with cell death signatures (autophagy, cuproptosis, ferroptosis, and pyroptosis signatures) [60–66]. The results showed that the C-index of our signature (0.7286131 in training sets, 0.6163404 in validation sets) surpassed that of other models in both the training and validation sets (Fig. 4K, L); our signature had better predictive performance for the estimating the prognosis of PDAC patients compared to other models.

### Prognostic independence analysis of hypoxia signature

**Fig. 5:**
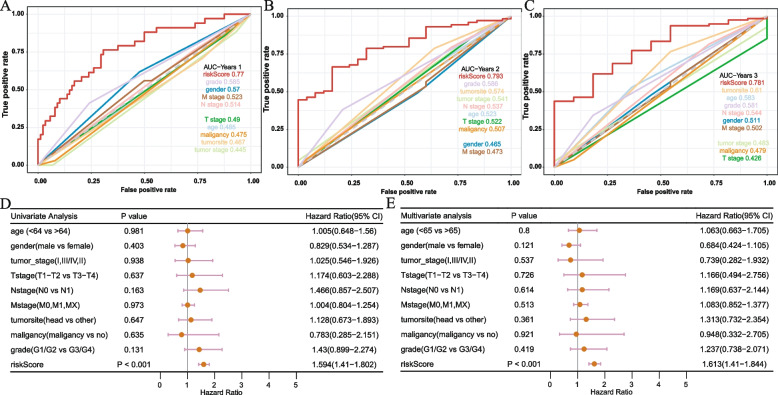
Clinical predictive evaluation of risk signature based on training sets (**A–C**) AUCs for risk score and clinical symptoms at 1, 2, and 3 years. **D, E** Univariate and multivariate Cox regression analysis of risk score and clinical feature

**Fig. 6:**
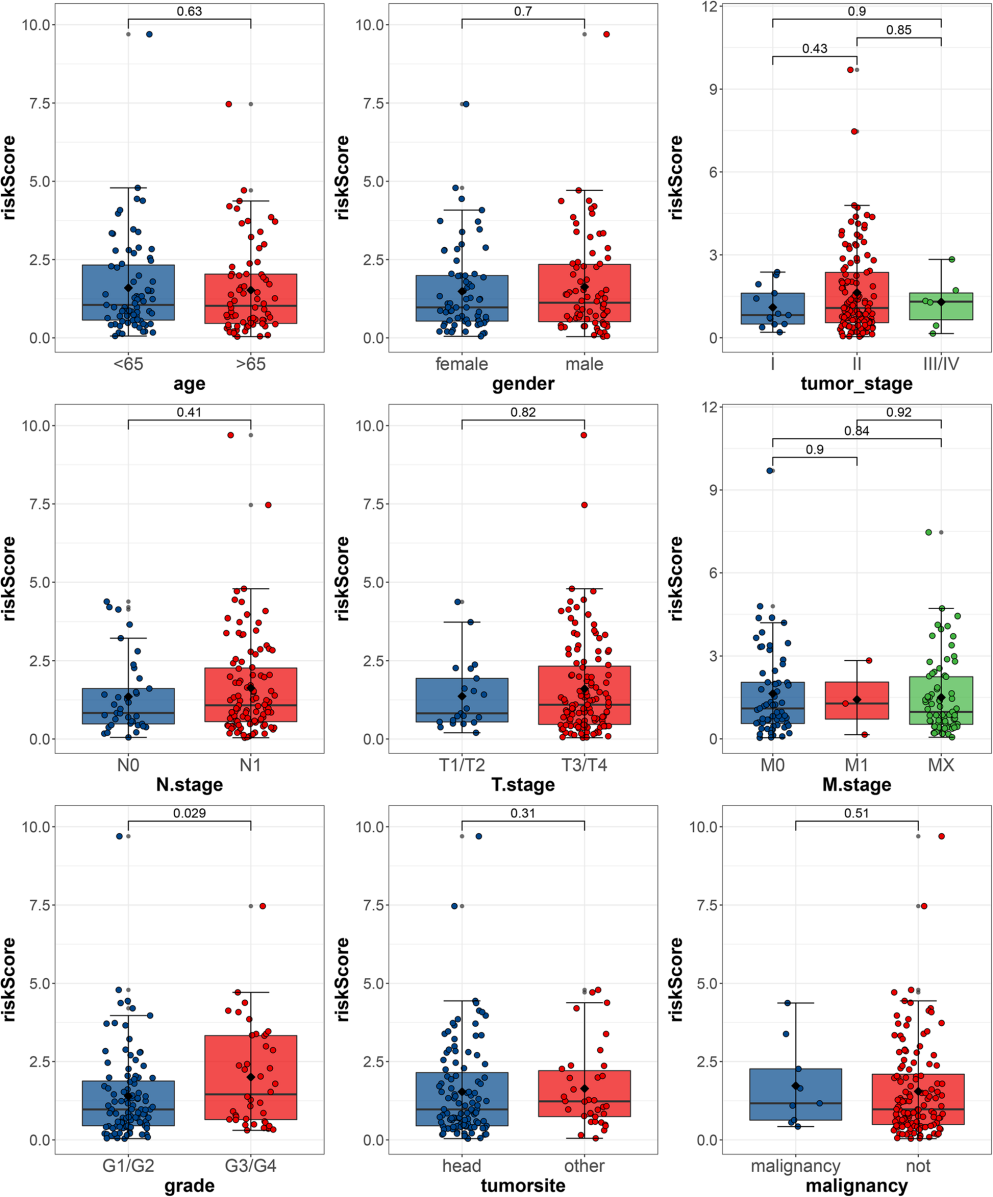
Association between prognostic risk signature and clinical symptoms The association between risk score and **A** age, **B** gender, **C** tumor stage, **D** N stage, **E** T stage, **F** M stage, **G** grade, **H** tumor site, and **I** malignancy. The number on the line is the *p*-value of Wilcoxon signed-rank sum test

To verify whether our signature can be used as an independent and effective prognostic indicator, we used the TCGA cohort to perform univariate and multivariate Cox regression analysis based on common clinical indicators, including age, gender, TNM stages, tumor site, malignancy, tumor stage, and tumor grade. The 1-, 2-, and 3-year AUC values of our signature were greater than those of other clinical predictors (Fig. 5A, B, C). Univariate and multivariate Cox regression results indicated that our signature was an independent prognostic indicator (Fig. 5D, E). We also analyzed the correlation between the risk score and different clinical factors, and the result indicated that our signature was statistically correlated with tumor grade (Fig. 6).

### Activation pathway and differentially expressed genes analysis between risk groups

**Fig. 7:**
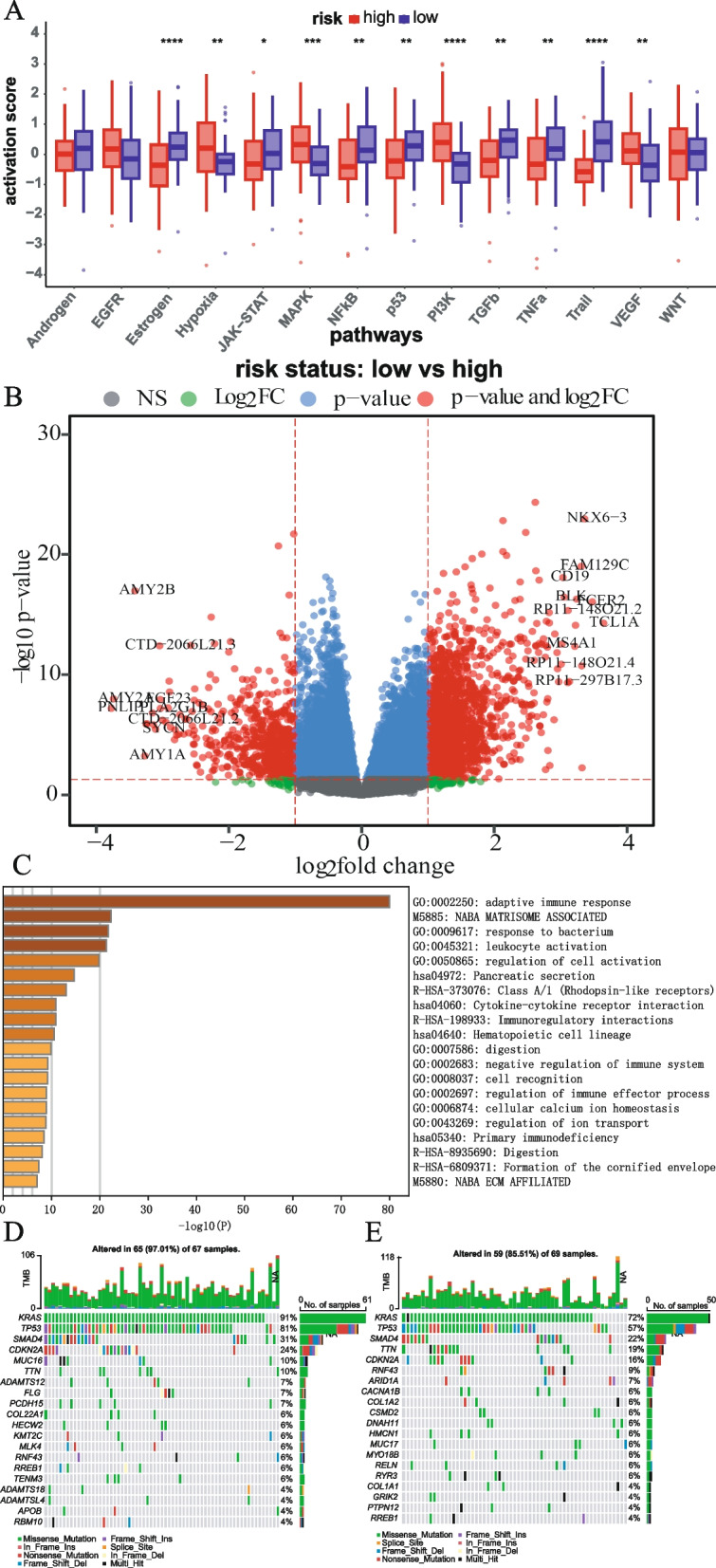
Analysis of differences in pathway activation and gene expression between risk groups. **A** Wilcoxon signed-rank test results of 14 typical pathway activation scores between high- and low-risk groups. **B** volcano plot of DEGs. **C** Pathway analysis results based on Metascape. **D, E** The mutation landscape of patients in the TCGA-PDAC cohort (show the top 20 genes) about the high- and low-risk groups (*p < 0.05, **p < 0.01, ***p < 0.001, ****p < 0.0001)

We conducted a comprehensive analysis to investigate the factors contributing to the different prognosis of patients in the high- and low-risk groups within the TCGA-PDAC dataset. Pathway activation analysis revealed significant activation of pathways associated with hypoxia tolerance, cell proliferation, promotion, angiogenesis promotion, and cell metastasis in the high-risk group (Fig. 7A) (such as Hypoxia, MARK, PI3K, and VEGF). Conversely, pathways related to immune response and surveillance, tumor inhibition and apoptosis induction, and trail were significantly activated in the low-risk group (Fig. 7A) (such as Estrogen, JAK-STAT, NFκB, P53, TGFβ, TGFα); 2057 DEGs between the high- and low-risk groups, with 547 downregulated and 1510 upregulated (Fig. 7B). Pathway enrichment analysis demonstrated that these DEGs were significantly enriched in the adaptive immune response pathway (Fig. 7C), suggesting potential differences in immune activation and response between the high- and low-risk groups. To visualize the common mutation frequencies of key genes, we presented a waterfall map depicting the top 20 genes with the highest mutation frequencies in the high- (Fig. 7D) and low-risk (Fig. 7E) groups. Notable, KRAS, TP53, SAMD4, CDKN2A, and TTN exhibited the highest mutation frequencies in both the high- and low-risk groups.

### Relationship between prognostic signature and tumor immune microenvironment

The proliferation and metastasis of malignant tumors are known closely influenced by the infiltration of immune cells within the tumor microenvironment. Given the enrichment analysis results suggesting potential differences in the activation of immune pathways between the high- and low-risk groups, we further explore the cancer immune microenvironment of the TCGA-PDAC dataset using various approaches.

**Fig. 8:**
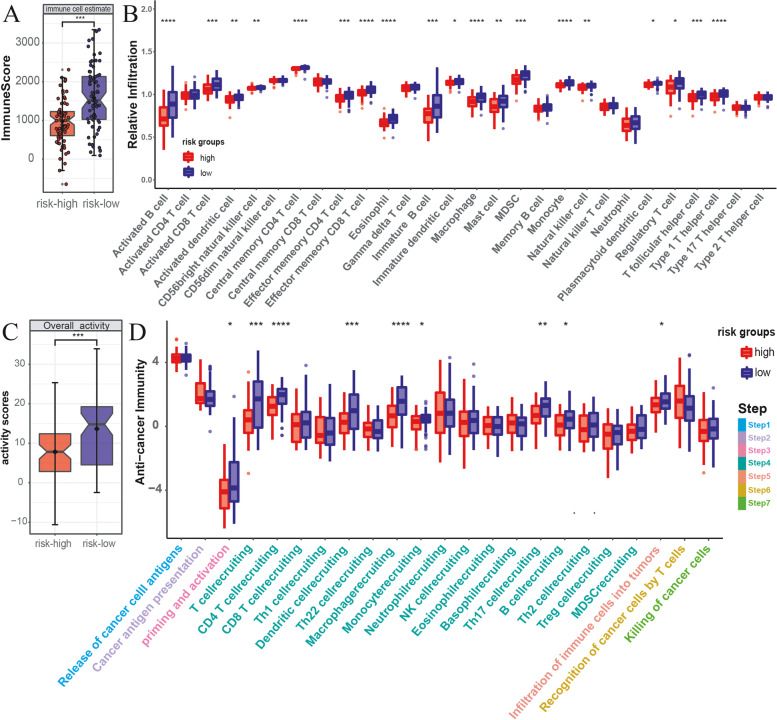
Analysis of tumor immune microenvironment. **A** Comparison of the immune scores of high- and low-risk groups in the TCGA-PDAC cohort. **B** Analysis of immune cell infiltration differences between the high- and low-risk groups. **C** Overall immune activation differences between high- and low-risk groups based on TIP. **D** Differences in the cancer immunity cycle activities between high- and low-risk groups (*p < 0.05, **p < 0.01, ***p < 0.001, ****p < 0.0001)

The analysis results obtained using the R package estimate indicated that the immune score, stromal score, and estimate score were significantly lower in the high-risk group (Fig. 8A, Fig. S4 A, B). Additionally, the tumor purity score was significantly higher in the high-risk group than that in the low-risk group (Fig. S4 C). The results of 28 kinds of immune cell infiltration degrees based on ssGSEA showed that the low-risk group had a more abundant immune cell composition. Specifically, immune cell types such as B cells, CD8 + T cells, eosinophil, macrophage, and monocyte exhibited significant differences between the high- and low-risk groups (Fig. 8B). To provide additional insights, we extensively analyzed the relationship between immune cell types and risk scores by integrating data from 7 mainstream immunoinformatics algorithms, and the results revealed that multiple immune cell types were negatively associated with risk scores (Fig. S5). We assessed the activation of the cancer immunity cycle, which reflects the degree of immune activation within the tumor microenvironment and predicts the functional performance of immune modulators and the chemokine system [45, 67]. The TIP results showed that overall immune activation was significantly higher in the low-risk group (Fig. 8C). Specifically, in steps such as priming and activation (step 3) and multiple cell recruiting factors (step 4), including T cell recruiting, B cell recruiting, CD4 T cell recruiting, dendritic cell recruiting, and macrophage recruiting, significant activation was observed in the low-risk group (Fig. 8D). However, there were no significant differences between the high- and low-risk groups in steps involving the release of cancer cell antigens (step 1), cancer antigen presentation (step 2), and subsequent steps related to the recognition and killing of cancer cells (steps 6 and 7) (Fig. 8D). The high activation of immune cell recruiting factors observed in the low-risk group may lead to increased immune cell infiltration and the maintenance of antitumor activity within the tumor microenvironment, thereby benefiting patients.

### Analysis of the predictive ability of hypoxia related signature for immunotherapy and chemotherapy

**Fig. 9:**
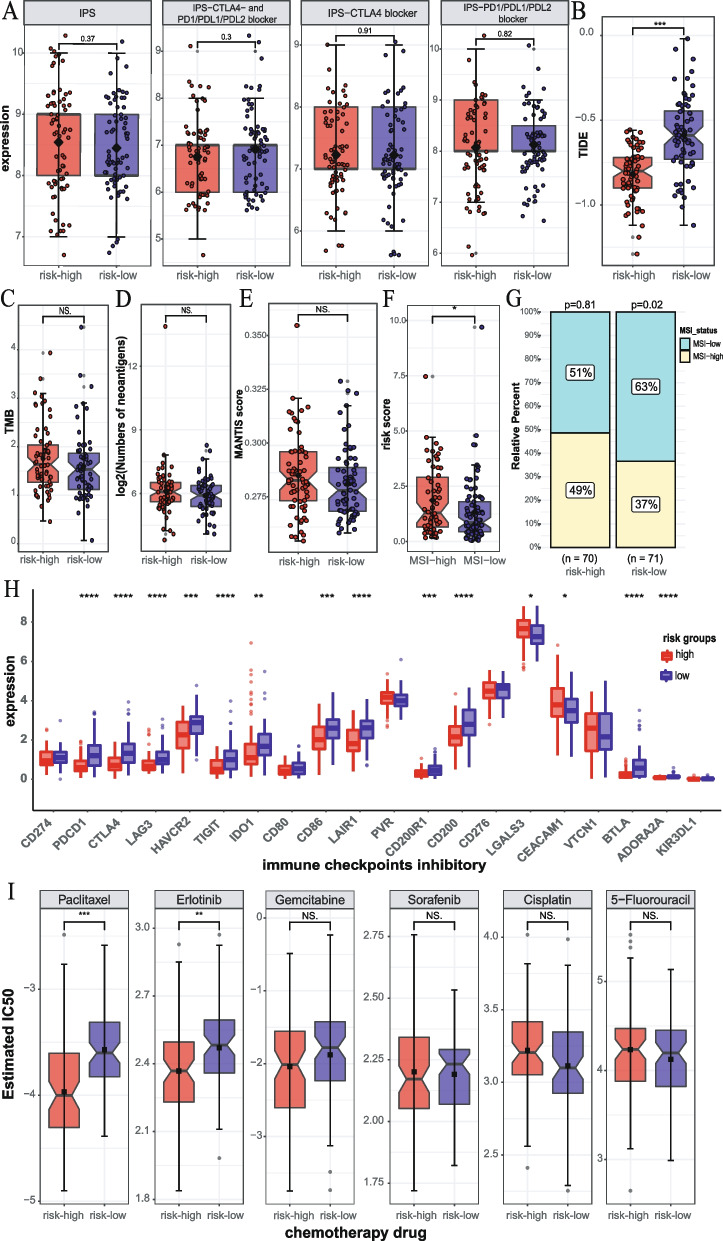
Analysis of the predictive ability of risk signature for immunotherapy and chemotherapy. **A–D** Association between IPS, TIDE, TMB, neoantigens, and the risk signature. **E** MSI difference analysis based on mantis between high- and low-risk groups. **F** Association analysis between MSI status (based on PreMSIm) and risk score. **G** Distribution analysis of MSI status in high- and low-risk groups. **H** The boxplot represents immune checkpoint expression in the high- and low-risk groups. **I** The difference in chemosensitivity of 6 commonly used anticancer drugs in the high- and low-risk groups (*p < 0.05, **p < 0.01, ***p < 0.001, ****p < 0.0001, NS. *p* > 0.05)

To evaluate the predictive ability of our signature for immunotherapy efficacy, we analyzed its association with several immunotherapy-related factors from multiple perspectives. IPS and TIDE have been verified to quantify the patients’ response to immune checkpoint inhibitors (ICIs). The results indicated that IPS, IPS-CTLA4- AND PD1/PDL1/PDL2 blocker, IPS-CTLA4 blocker, and IPS-PD1/PDL1/PDL2 blocker scores showed no significant association with the risk score (Fig. 9A). However, the TIDE analysis revealed that the TIDE score was significantly higher in the low risk-group compared to the high-risk group (Fig. 9B). It is worth noting that all patients had TIDE score below 0, suggesting that patients in the low-risk group may not respond significantly better to immunotherapy than those in the high-risk group. TMB, neoantigens, and MSI are common predictors of immunotherapy efficacy [14, 68, 69]. The results showed that TMB and neoantigens had no significant correlation with risk scores (Fig. 9C, D). Then, we used mantis and PreMSIm to predict MSI levels in TCGA-PDAC patients and analyzed their correlation with risk scores. The results of mantis showed that MSI had no significant correlation with risk scores (Fig. 9E). PreMSIm showed that MSI status had a significant correlation with risk scores, and the number of MSI-low patients in the low-risk group was significantly higher than that in the high-risk group (Fig. 9F, G). These results indicate that this signature is difficult to predict the efficacy of patients’ ICIs, but the prediction bias caused by the poor effect of current ICIs on PDAC is also noteworthy. So, we also investigated the relationship between the expression of 20 ICIs with therapeutic potential and risk scores and found that the risk score was negatively correlated with most ICIs, including PD-L1, CTLA-4, LAG-3, TIM-3, IDO1, TIGIT, CD86, LAIR1, CD200R1, and CD200, while it was positively correlated with LGALS3 and CEACAM1 (Fig. 9H, Fig. S6). Although the expression of ICIs differs between the high- and low-risk groups, the low expression of ICIs may also contribute to the poor effectiveness of PDAC immunotherapy. Finally, we analyzed the difference in chemosensitivity to 6 commonly used anticancer drugs between the high- and low risk groups, and the results showed that the IC50 values of paclitaxel and erlotinib were relatively higher in the low-risk group (Fig. 9I).

### Functional analysis of risk genes

**Fig. 10:**
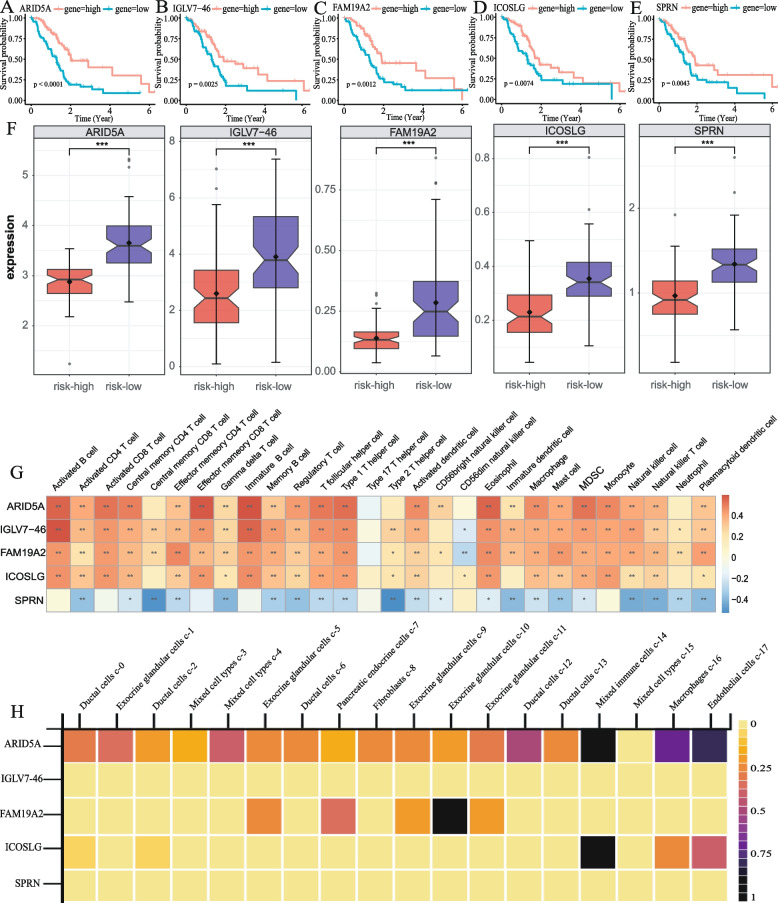
Functional analysis of risk genes. **A–E** Kaplan–Meier curve of OS in PDAC patients with high- and low-expression of 5 genes. **F** Expression patterns of 5 genes in the high- and low-risk groups. **G** Correlation matrix of the relationship between the expression of 5 risk genes and the differential immune infiltration levels. **H** Expression of risk genes in different cell types based on HPA database (*p < 0.05, **p < 0.01, ***p < 0.001, ****p < 0.0001)

We conduced survival analysis of the five risk genes and found that patients with high expression of these genes had a better prognosis (Fig. 10A–E). Consistently, these genes were significantly upregulated in the low-risk group (Fig. 10F), indicating that they act as protective factors. We then analyzed the association of the risk genes with the 28 immune cell types, and the results revealed that ARID5A, IGLV7-46, FAM19A2, and ICOSLG were significantly positively correlated with multiple immune cells, while SPRN showed a negative correlation with multiple immune cells (Fig. 10G), suggesting a correlation between our signature and the tumor immune microenvironment. Single-cell level gene expression analysis of the HPA database showed that ARID5A was significantly expressed in mixed immune cells, macrophages, and endothelial cells, and the expression level of ARID5A was generally higher than other risk genes in a various of cells (Fig. 10H).

**Fig. 11:**
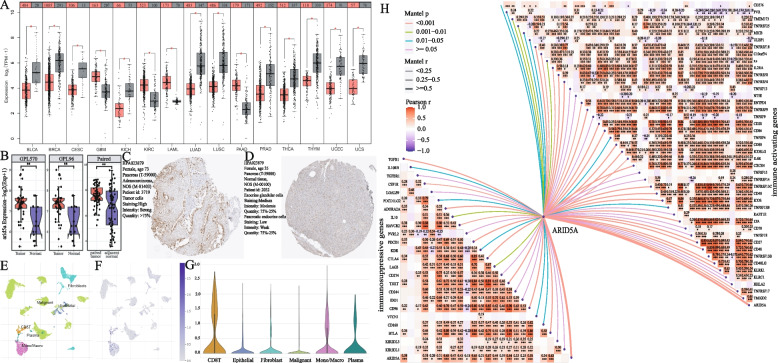
Expression and function analysis of ARID5A. **A** Pan-cancer expression analysis of ARID5A, red represents tumor samples and gray represents normal samples. **B** Expression analysis of ARID5A based on GEO chip data, the first and second are the expression level of ARID5A in unpaired samples based on GPL570 and GPL96 platforms, and the last on represents is the expression level of ARID5A based on paired samples. **C, D** Immunohistochemical staining of ARID5A in pancreatic cancer tumor tissues (**C**) and normal tissues (**D**). **E–G** Expression of ARID5A at the cellular level based on the GSE154778 dataset. **H** Correlation between ARID5A and the immune activating genes or immunosuppressive genes (*p < 0.05, **p < 0.01, ***p < 0.001, ****p < 0.0001)

The results of multivariate Cox regression analysis highlighted the significant impact of ARIT5A on the prognosis of PDAC (*p* < 0.001, hazard ratio < 1) (Fig. 3E). Subsequently, we conducted several analyses to explore the function of ARID5A in PDAC. Pan-cancer expression analysis revealed that ARID5A was significantly overexpressed in tumor tissues of GBM, KIRC, LAML, and PAAD, while it showed lower expression in tumor tissues of BLCA, BRCA, and CESC (Fig. 11A). Survival analysis showed that high expression of ARID5A was beneficial to the prognosis of BRCA, CESC, HNSC, LUAD, and SKCM (Fig. S7A-E), but unfavorable for GBMLGG, KIRC, KIRP, LUSC, READ, STAD, and UCEC (Fig. S7F-L). Furthermore, gene expression analysis based on chip datasets in GEO demonstrated that ARID5A was significantly highly expressed in tumor tissues (paired or unpaired samples, Fig. 11B). Protein expression analysis also revealed significantly higher levels of ARID5A in tumor tissues (Fig. 10C, D). Single-cell analysis of the GSE154778 dataset in the TISCH database showed a predominant expression of ARID5A in CD8T cells (Fig. 11E, F, G). Gene expression correlation analysis further highlighted the closely relationship between ARID5A and various immunoactivator or suppressor genes (Fig. 11H).

## Discussion

PDAC is a highly lethal malignancy [1]. Due to rapid progression, easy metastasis and high drug resistance of PDAC, little progress has been made in its treatment [3, 70, 71]. Therefore, there is a critical need to identify effective prognostic biomarkers and novel therapeutic targets for PDAC patients. With the increasing utilization of high-throughput sequencing in cancer research, recent studies have sought to construction gene expression-based signatures to assess the prognosis of patients with various malignant tumors [72–74].

Hypoxia plays a significant role in promoting the proliferation, metastasis, and therapy resistance to radiotherapy and chemotherapy of PDAC [19, 23, 75]. However, direct measurement of tumor hypoxia status is challenging, and researchers often rely on gene expression profiles to predict hypoxia status in tumors [76, 77]. Our clustering analysis revealed a high mortality cohort (cluster_2) associated with hypoxia in PDAC patients. Cluster_2 exhibited higher activation of hypoxia pathway and worse prognosis, consistent with previous studies linking high hypoxia levels to poor outcomes [78]. The regulation of hypoxia on the tumor microenvironment is complex, and genes differentially expressed in relation to hypoxia may not fully capture the molecular mechanisms. Therefore, we utilized WGCNA to analyze gene sets associated with hypoxia, and our GO and KEGG pathway revealed the enrichment of several immunoregulatory pathways. Previous research has suggested that hypoxia is a barrier to PDAC immunotherapy [79]. Our findings further support the close relationship between hypoxia and immunity in PDAC.

Among the five signature genes (ARID5A, IGLV7-46, FAM19A2, ICOSLG, SPRN) identified through LASSO and multivariate Cox regression analysis, ARID5A stands out as a member of the ARID protein family with crucial roles in development, cell growth regulation, and immune regulation [80, 81]. Recent studies highlighted the regulatory role of ARID5A in various cancers, including lung, breast, prostate, PDAC, and colorectal cancer [82–85]. Notably, Parajuli et al. demonstrated that high expression of ARID5A contributes to tumor immune evasion by recruiting immunosuppressive cells, thereby reducing the recruitment and activation of antitumor effector T cell within the tumor microenvironment [85]. According to our results, the expression of ARID5A is positively associated with most immune cells (like CD8T, Mono/Macro cells) and was also significantly associated with multiple immunomodulatory genes. The expression of ARID5A is related to the infiltration of immune cells, and the highly active immune pathway is conducive to the survival prognosis of patients. Therefore, high expression of ARID5A in tumor tissue may serve as a potential target for PDAC immunotherapy. Interestingly, pan-cancer analysis revealed different prognostic implications of ARID5A in distinct cancer subtypes within the same organ. While ARID5A showed high expression in normal tissues of both LUAD and LUSC, survival analysis indicated that high expression of this gene was beneficial to the prognosis of LUAD but detrimental to LUSC. Thus, the molecular mechanisms underlying the regulation of ARID5A in tumor could be of interest in future studies.

IGLV7-46 is an immunoglobulin lambda variable gene. While the specific role of IGLV7-46 in PDAC or tumor immunity remains to be elucidated, Cai et al. have speculated on a potential correlation between FAM19A2 and immune response based on data mining [86]. One the other hand, ICOSLG has been established as an immune checkpoint molecule that is closely associated with immune activation and tumor progression. It has been implicated ICOSLG as as a predictor and therapeutic target in acute lymphoblastic leukemia, and can promote the progression of glioblastoma by mediating the regulatory T-cell expansion [87, 88]. As for SPRN, its function in the context of PDAC or tumor immunity has not been reported, and further research is needed. Overall, the risk genes identified in our signature exhibit close associations with tumor immunity, highlighting the interplay between tumor hypoxia and immune activation.

Several prognostic signatures for pancreatic cancer based on gene sets, such as hypoxia, pyroptosis, ferroptosis, cuproptosis, and autophagy, have been published recently [60–66]. Our analysis revealed that the C-index value of our signature was higher than or comparable to the published prognostic signatures based on the hypoxia gene set, both in the training and validation sets. To our knowledge, this present study is the first to utilize WGCNA to analyze and fully incorporate gene sets that are significantly associated with hypoxia to construct the prognostic model. Overall, our prognostic signature demonstrates superior or comparable performance to previously defined signatures in pancreatic cancer.

The pathway activation analysis demonstrated that pathways associated with cell proliferation and angiogenesis were significantly activated in the high-risk group, while pathways related to tumor suppression, immune response, and apoptosis were significantly activated in the low-risk group. These findings are consistent with the observed poor prognosis in the high-risk group and better prognosis in the low-risk group. The activity and infiltration of immune cells in the tumor microenvironment play a critical role in tumor pathogenesis and progression. PDAC is known for its immunosuppressive nature and low immunogenicity, making it highly aggressive [89]. The pathway enrichment analysis of DEGs revealed significant differences in the adaptive immune response pathway between the high- and low-risk groups. The estimate analysis further supported these findings, showing higher immune scores and stromal scores in the low-risk group, while the tumor purity score showed the opposite trend. The TIP analysis indicated that overall immune activation and infiltration of immune cells into tumors were significantly higher in the low-risk group. Taking these results together, it can be speculated that the poor prognosis in the high-risk group may attribute to lower immune activation and infiltration in the tumor microenvironment.

The immunocyte enrichment analysis revealed that the immune microenvironment of the low-risk group is complex and exhibits contradictory characteristics. CD8 + T cells were found to inhibit tumor growth in different ways [90] and significantly enriched in low-risk groups. Dendritic cells, which play a role in inducing and regulating antitumor immune responses [91], were also significantly recruited in the low-risk group. CD4 + T cells, which have been associated associated with metastasis and reduced survival in some studies [92], were unexpectedly enriched in the low-risk group. Myeloid derived suppressor cells (MDSCs), known for their immunosuppression and immune evasion promoting properties [93], were significantly enriched in the low-risk group. Macrophages, which have been linked to immune suppression and poor prognosis at high levels [94, 95], were also found to be significantly enriched in the low-risk group. Eosinophils and mast cells, which have been implicated pancreatic fibrosis and malignancy development [96], showed significant enrichment in the low-risk group. These findings indicate that the tumor microenvironment heterogeneity among patients in the low-risk group could be more pronounced compared to the high-risk group.

Immune checkpoint blockade therapy is not effective in patients with PDAC. Our analysis also found no significant difference in immunotherapy outcomes between the high- and low-risk groups. Related studies speculated that mainstream immune checkpoints such as PD1, PDL1, and CTLA4 might not be the main immunosuppressive molecules of PDAC [97]. The combination of immunocheckpoint inhibitors and chemotherapy showed good benefits for PDAC patients [71]. Therefore, combined with the results of our analysis of immune checkpoints and chemotherapy agents, our results can provide referential treatment options for patients in different risk groups.

Early diagnosis plays a crucial role in improving cancer treatment outcomes, and the risk genes identified in our study have the potential to contribute to early diagnosis of PDAC. The analysis of ARID5A expression in pan-cancers and immunohistochemical results indicate its high expression in PDAC. Single-cell expression analysis further reveals its high expression in CD8T cells. Additionally, its expression level has been correlated with the prognosis of various cancers, highlighting its potential as a diagnostic biomarker for PDAC.

However, it is important to acknowledge the limitations of our study. Firstly, the lack of validation in real-world clinical cohorts and the small number of patients in the validation study restrict the generalizability of our findings. Secondly, the expression of risk genes lacks experimental validation in clinical samples or at the cellular level, such as through real-time PCR. These limitations should be addressed in future research to further validate the diagnostic potential of the identified risk genes in PDAC.

## Conclusion

In conclusion, this study introduces a hypoxia-based prognostic molecular signature for PDAC and explores the underlying factors contributing to prognostic differences in high- and low-risk groups based on this signature. The high-risk group, characterized by high hypoxia and poor prognosis, exhibits a typical immune cold phenotype, whereas the low-risk group demonstrates a favorable prognosis and extensive immune cell infiltration, offering new insights into PDAC treatment. The identified signature holds potential as a prognostic marker for PDAC and can inform the combination of immunotherapy and chemotherapy. Among the risk genes, ARID5A emerges as promising therapeutic target and a potential molecular marker for early diagnosis of PDAC.

### Supplementary Information

**Additional file 1:** **Figure S1.** Wilcoxon signed-rank test results of 14 typical pathway activation scores between cluster_1 and cluster_2 groups. (*p<0.05, **p<0.01, ***p<0.001, **** *p*<0.0001).

**Additional file 2:** **Figure S2.** Scatter plots of the module membership (MM) and gene significance (GS) of each gene in 8 modules which significantly associated with hypoxia cluster.

**Additional file 3:** **Figure S3.** Results of KEGG pathway analysis of candidate gene set.

**Additional file 4:** **Figure S4.** Comparison of the stromal, estimate and tumor purity scores of high- and low-risk groups in the TCGA-PDAC cohort. (*p<0.05, **p<0.01, ***p<0.001, **** *p*<0.0001).

**Additional file 5:** **Figure S5.** Spearman correlation analysis about risk score and tumor-infiltrating immune cells.

**Additional file 6:** **Figure S6.** Correlation between risk score and 20 inhibitory immune checkpoints. The color and the values indicate the Spearman correlation coefficient.

**Additional file 7:** **Figure S7.** Pan-cancer survival analysis of ARID5A.

## Data Availability

The mRNA-seq transcriptome profiling and corresponding clinical data of PAC patients for the training set (TCGA database, https://portal.gdc.cancer.gov/repository) and validation set (ICGC database, https://dcc.icgc.org/) were downloaded from UCSC Xena (https://xenabrowser.net/datapages/).

## Abbreviations

PDAC: Pancreatic ductal adenocarcinoma
WGCNA: Weighted gene co-expression network analysis
LASSO: Least absolute shrinkage and selection operator
ICGC: International Cancer Genome Consortium
OS: Overall survival
AJCC: American Joint Committee on Cancer
TNM: Tumor-node-metastasis
ICIs: Immune checkpoint inhibitors
PD-L1: Programmed death-ligand 1
TMB: Tumor mutation burden
NAL: Neoantigen load
MSI-H: Microsatellite instability-high
FPKM: Fragments per kilobase of exon per million reads mapped
MsigDB: Molecular Signatures Database
GDC: Genomic Data Commons
CDF: Cumulative distribution function
K-M: Kaplan–Meier
Mes: Module eigengenes
KEGG: Kyoto Encyclopedia of Genes and Genomes
GO: Gene Ontology
ROC: Receiver operating characteristic curves
C index: Concordance index
DEGs: Differentially expressed genes
FC: Fold change
ssGSEA: Single-sample gene set enrichment analysis
TIP: Tracking tumor Immunophenotype
IPS: Immunophenotype
TCIA: Cancer Immunome Atlas
IC50: Half-maximal inhibitory concentration
GDSC: Genomics of Drug Sensitivity in Cancer
HPA: The Human Protein Atlas
GEPIA2: Gene Expression Profiling Interactive Analysis
GENT2: Gene Expression database of Normal and Tumor tissues
TNMplot: The differential expression analysis in Tumor, Normal, and Metastatic tissues
