# Supplementary figures and images for: Novel prognostic gene signature for pancreatic ductal adenocarcinoma based on hypoxia
Source: World J Surg Oncol. 2023 Aug 22;21:257. doi: 10.1186/s12957-023-03142-2 (PMC10464224; doi:10.1186/s12957-023-03142-2)

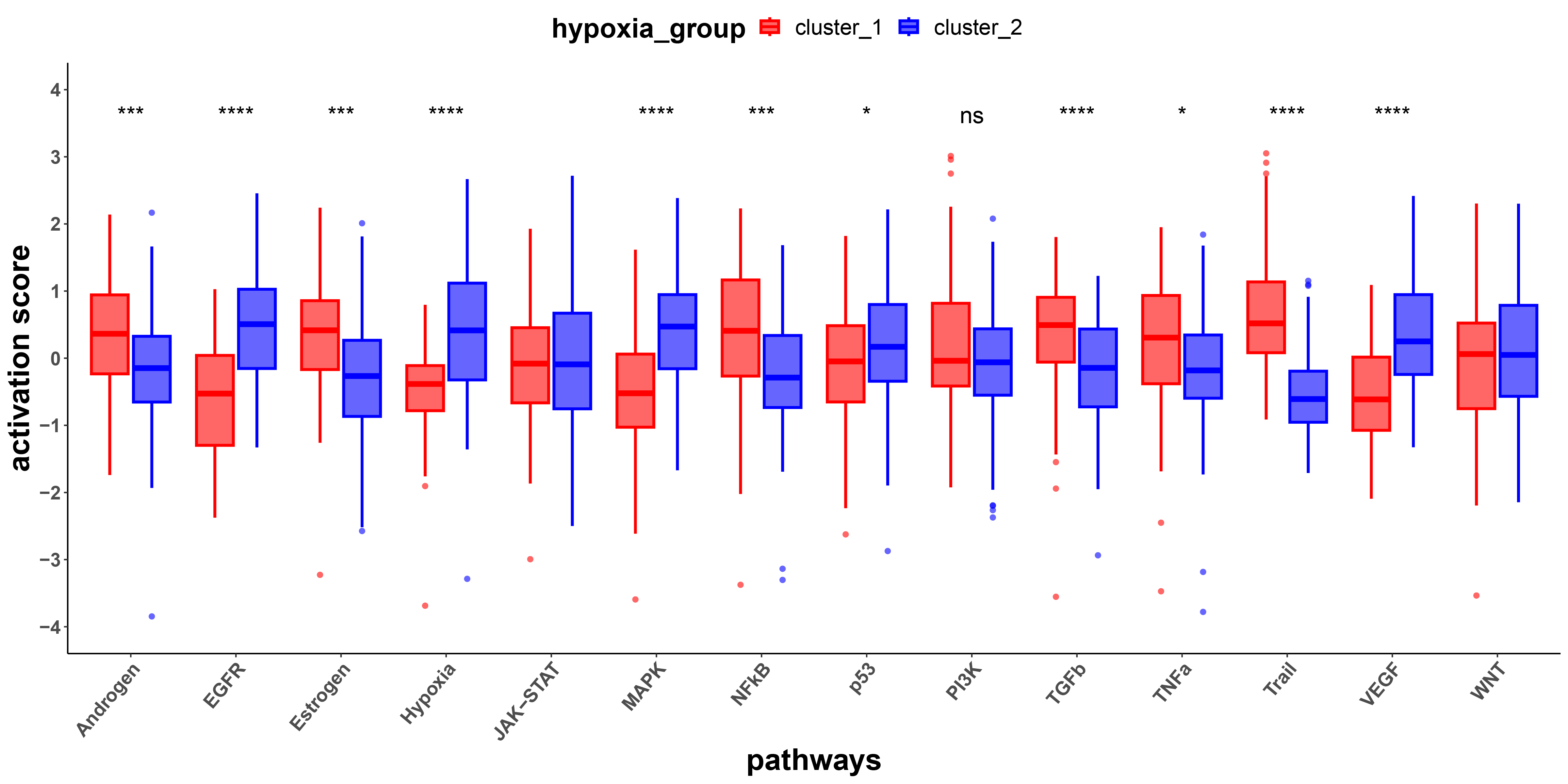

Supplement: Supplementary file 1 — Additional file 1: Figure S1. Wilcoxon signed-rank test results of 14 typical pathway activation scores between cluster_1 and cluster_2 groups. (*p<0.05, **p<0.01, ***p<0.001, **** p<0.0001). [file 12957_2023_3142_MOESM1_ESM.tif]

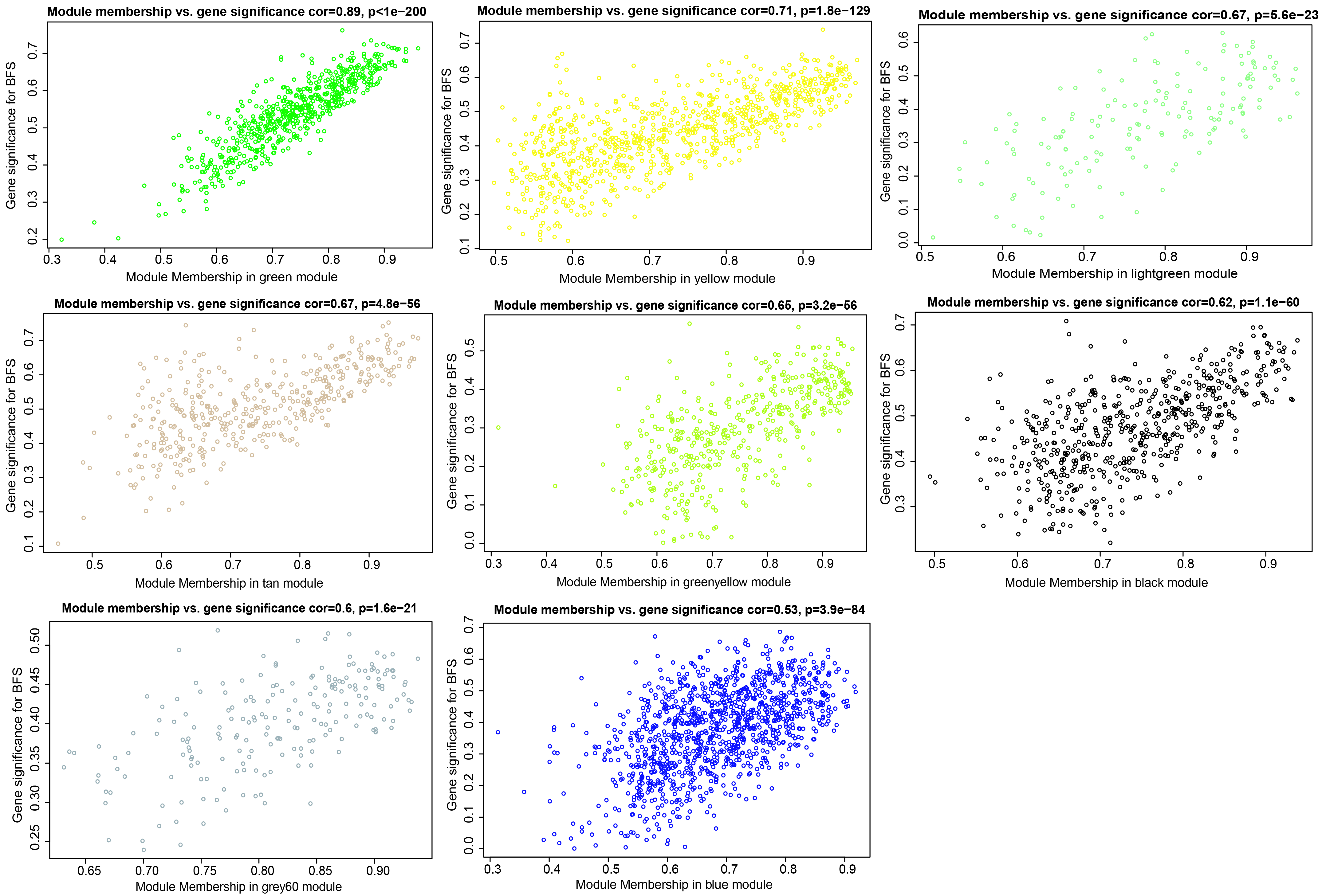

Supplement: Supplementary file 2 — Additional file 2: Figure S2. Scatter plots of the module membership (MM) and gene significance (GS) of each gene in 8 modules which significantly associated with hypoxia cluster. [file 12957_2023_3142_MOESM2_ESM.tif]

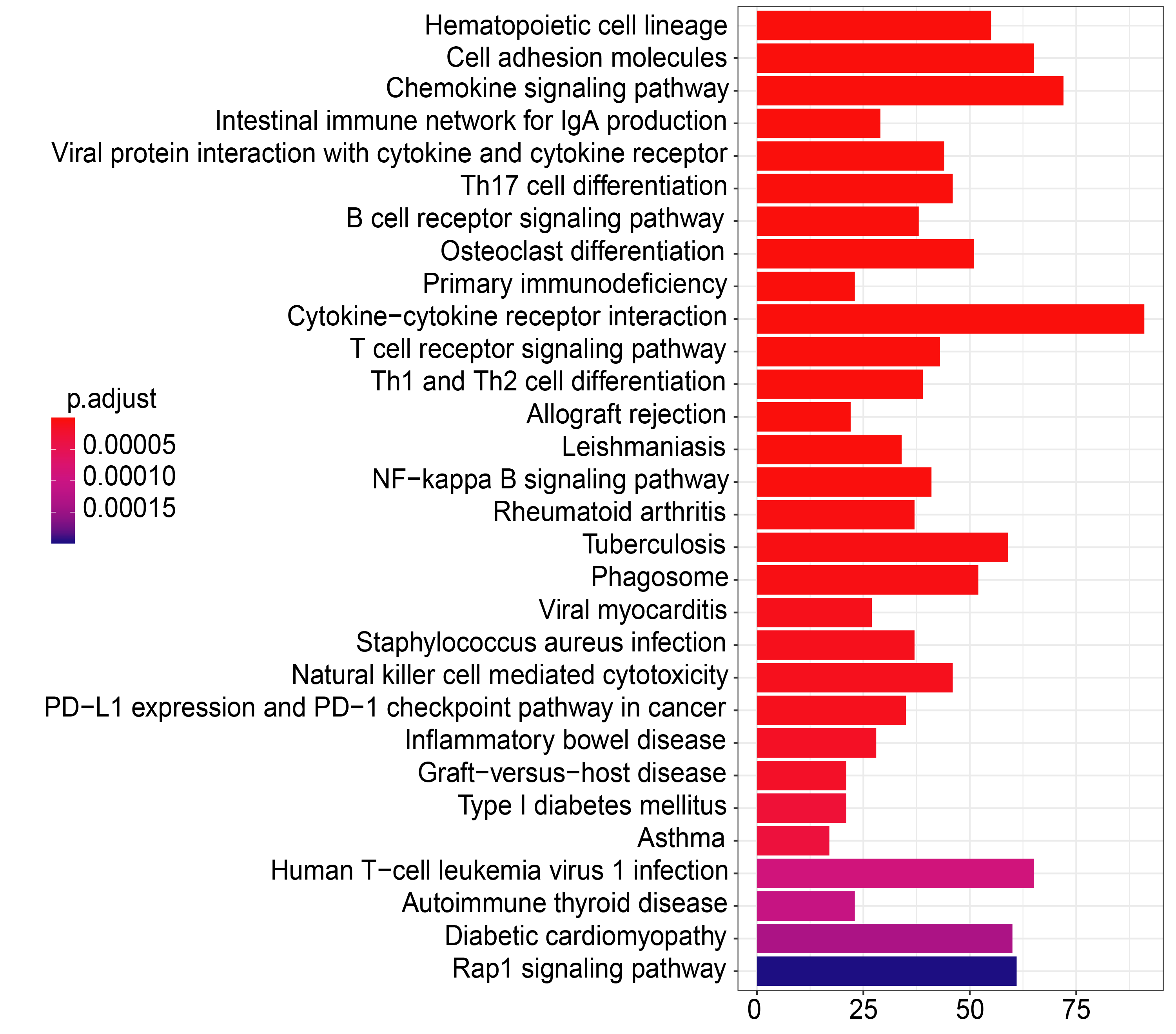

Supplement: Supplementary file 3 — Additional file 3: Figure S3. Results of KEGG pathway analysis of candidate gene set. [file 12957_2023_3142_MOESM3_ESM.tif]

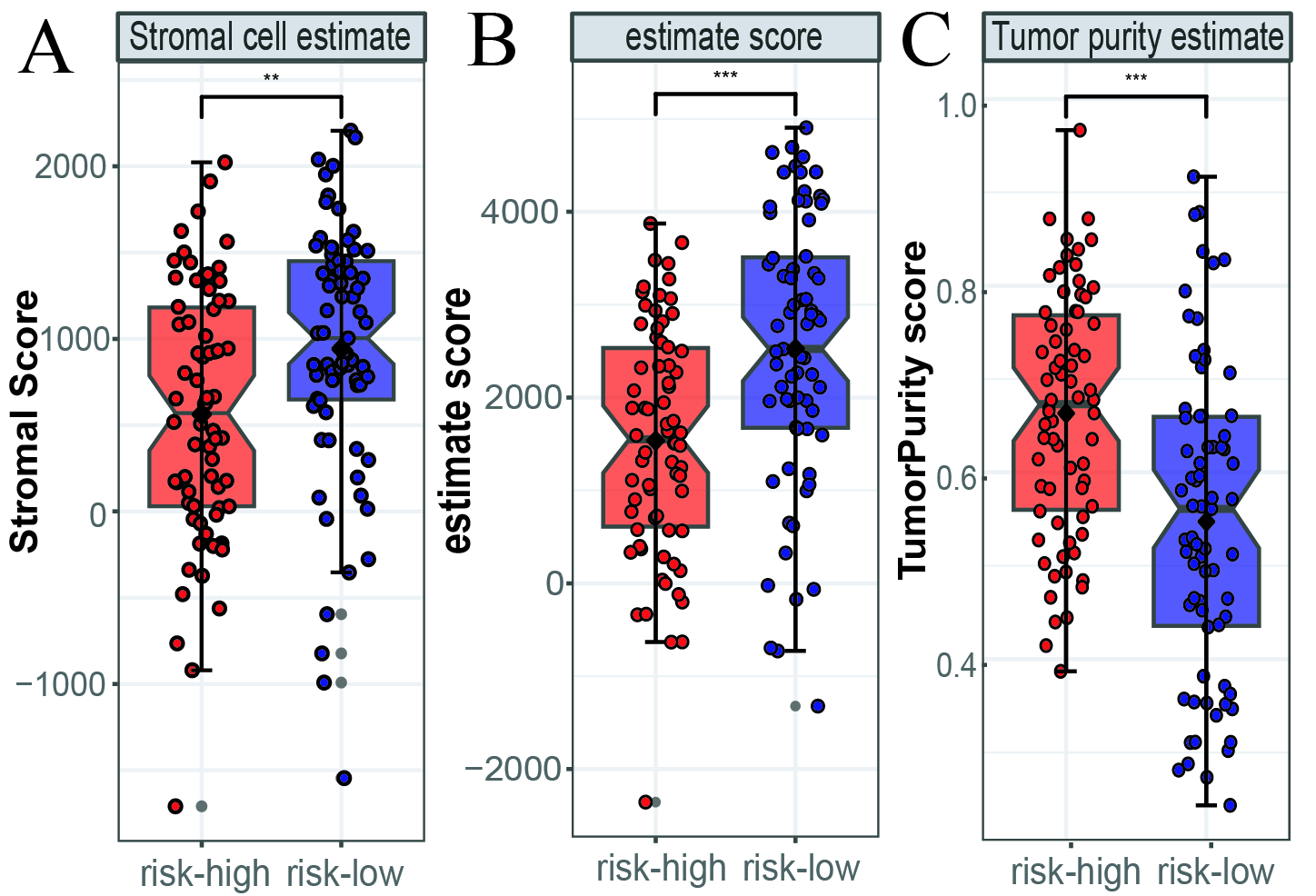

Supplement: Supplementary file 4 — Additional file 4: Figure S4. Comparison of the stromal, estimate and tumor purity scores of high- and low-risk groups in the TCGA-PDAC cohort. (*p<0.05, **p<0.01, ***p<0.001, **** p<0.0001). [file 12957_2023_3142_MOESM4_ESM.tif]

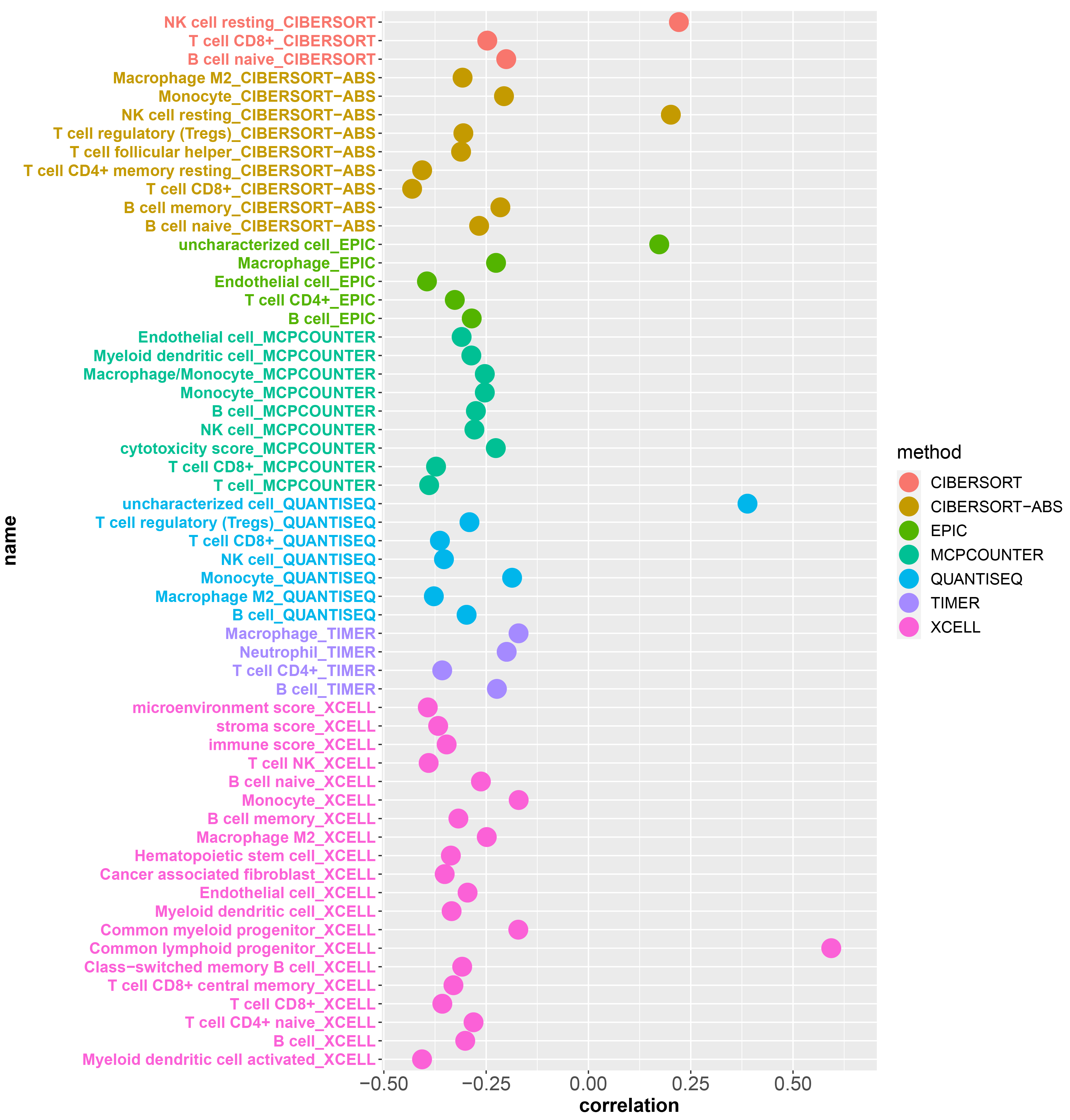

Supplement: Supplementary file 5 — Additional file 5: Figure S5. Spearman correlation analysis about risk score and tumor-infiltrating immune cells. [file 12957_2023_3142_MOESM5_ESM.tif]

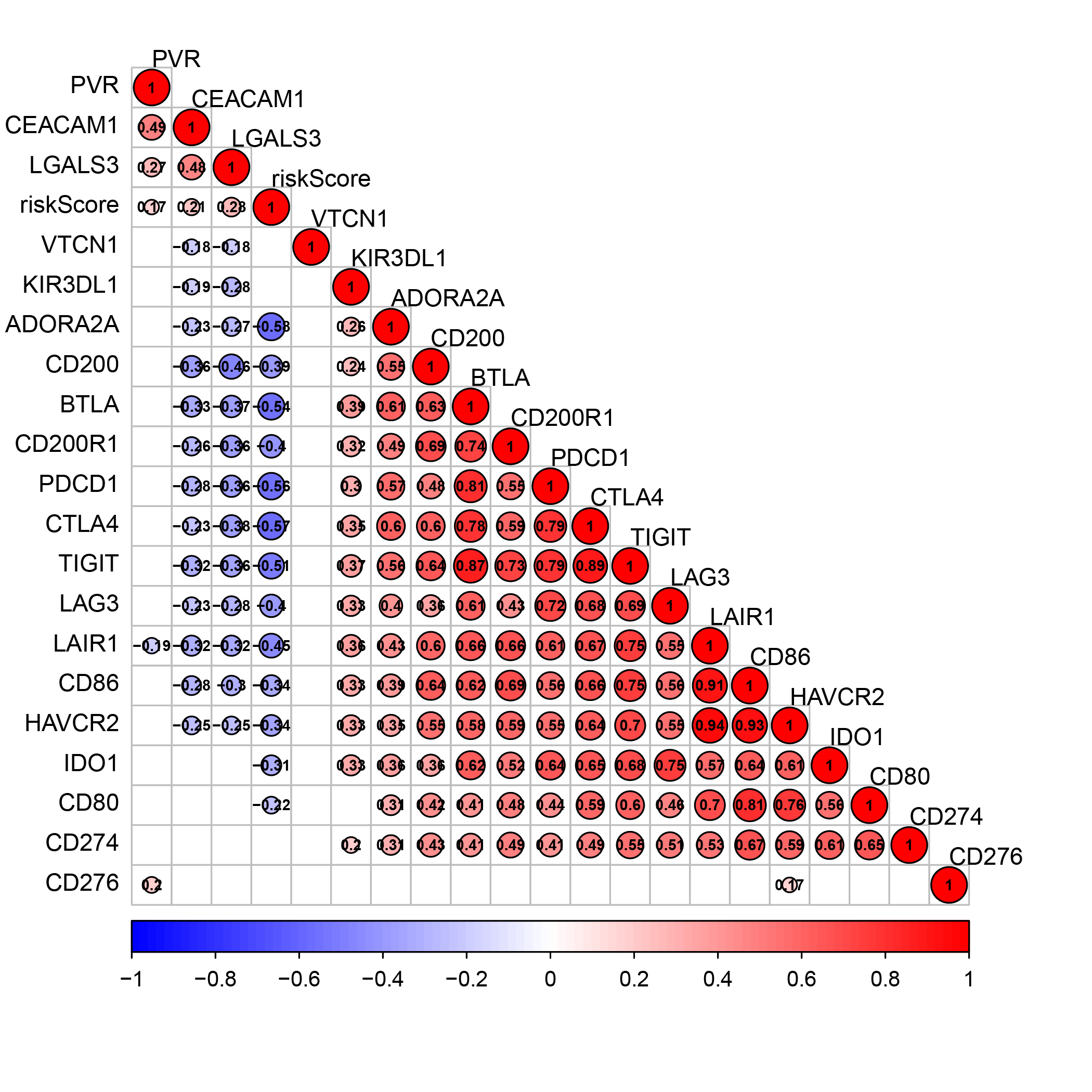

Supplement: Supplementary file 6 — Additional file 6: Figure S6. Correlation between risk score and 20 inhibitory immune checkpoints. The color and the values indicate the Spearman correlation coefficient. [file 12957_2023_3142_MOESM6_ESM.tif]
